# Supplementary material for: Identification and functional analysis of non-coding regulatory small RNA FenSr3 in Bacillus amyloliquefaciens LPB-18
Source: PeerJ. 2023 May 15;11:e15236. doi: 10.7717/peerj.15236 (PMC10194069; doi:10.7717/peerj.15236)
Supplement: Supplemental Information 4 [file peerj-11-15236-s004.zip › KO/CK-vs-T1_map/map04725.html]

KEGG PATHWAY: Cholinergic synapse - Reference pathway


|  |  |
| --- | --- |
| **Cholinergic synapse - Reference pathway** |  |

[
Pathway menu
| Organism menu
| Pathway entry
| Show description
| User data mapping
]

Acetylcholine (ACh) is a neurotransmitter widely distributed in the central (and also peripheral, autonomic and enteric) nervous system (CNS). In the CNS, ACh facilitates many functions, such as learning, memory, attention and motor control. When released in the synaptic cleft, ACh binds to two distinct types of receptors: Ionotropic nicotinic acetylcholine receptors (nAChR) and metabotropic muscarinic acetylcholine receptors (mAChRs). The activation of nAChR by ACh leads to the rapid influx of Na+ and Ca2+ and subsequent cellular depolarization. Activation of mAChRs is relatively slow (milliseconds to seconds) and, depending on the subtypes present (M1-M5), they directly alter cellular homeostasis of phospholipase C, inositol trisphosphate, cAMP, and free calcium. In the cleft, ACh may also be hydrolyzed by acetylcholinesterase (AChE) into choline and acetate. The choline derived from ACh hydrolysis is recovered by a presynaptic high-affinity choline transporter (CHT).

|  |  |  |
| --- | --- | --- |
| Reference pathway | 184% 150% 122% 100% 82% 67% 55% | 图片下载 |
